# Comparative Analysis of Glycoprotein B (gB) of Equine Herpesvirus Type 1 and Type 4 (EHV-1 and EHV-4) in Cellular Tropism and Cell-to-Cell Transmission

**DOI:** 10.3390/v7020522

**Published:** 2015-02-03

**Authors:** Bart Spiesschaert, Nikolaus Osterrieder, Walid Azab

**Affiliations:** 1 Institut für Virologie, Robert von Ostertag-Haus, Zentrum für Infektionsmedizin, Freie Universität Berlin, Robert-von-Ostertag-Str. 7-13, Berlin 14163, Germany; E-Mails: bart.spiesschaert@fu-berlin.de (B.S.); no.34@fu-berlin.de (N.O.); 2 Department of Virology, Faculty of Veterinary Medicine, Zagazig University, Zagazig 44519, Egypt

**Keywords:** EHV-1, glycoprotein B, tropism, equine

## Abstract

Glycoprotein B (gB) plays an important role in alphaherpesvirus cellular entry and acts in concert with gD and the gH/gL complex. To evaluate whether functional differences exist between gB1 and gB4, the corresponding genes were exchanged between the two viruses. The gB4-containing-EHV-1 (EHV-1_gB4) recombinant virus was analyzed for growth in culture, cell tropism, and cell entry rivaling no significant differences when compared to parental virus. We also disrupted a potential integrin-binding motif, which did not affect the function of gB in culture. In contrast, a significant reduction of plaque sizes and growth kinetics of gB1-containing-EHV-4 (EHV-4_gB1) was evident when compared to parental EHV-4 and revertant viruses. The reduction in virus growth may be attributable to the loss of functional interaction between gB and the other envelope proteins involved in virus entry, including gD and gH/gL. Alternatively, gB4 might have an additional function, required for EHV-4 replication, which is not fulfilled by gB1. In conclusion, our results show that the exchange of gB between EHV-1 and EHV-4 is possible, but results in a significant attenuation of virus growth in the case of EHV-4_gB1. The generation of stable recombinant viruses is a valuable tool to address viral entry in a comparative fashion and investigate this aspect of virus replication further.

## 1. Introduction

Glycoprotein B (gB) is a type 1 transmembrane protein and represents a highly conserved class III fusion protein present in members of the *Herpesviridae* family [1]. In members of the *Alphaherpesvirinae*, it is thought that gB mediates the virus entry process through membrane fusion after initial attachment of the virion via gC to cell surface glycosaminoglycans, binding of gD to its cognate receptor and activation of the heterodimeric gH-gL complex, which in turn primes gB for fusion [2,3,4,5,6,7,8,9]. In Herpes simplex virus type 1 (HSV-1), gB has been shown to bind several cellular receptors to facilitate viral entry; however, there is no data available on the role of equine herpesvirus type 1 (EHV-1) gB in receptor binding [1]. HSV-1 gB can bind to the paired immunoglobulin-like type 2 receptor (PILRα) to trigger viral fusion in the presence of gD [10]. In addition to PILRα, non-muscle myosin heavy chain IIA (NMMHCIIA; also known as myosin 9) [11] and myelin-associated glycoprotein (MAG) [12], a protein expressed in neuronal tissues, were also shown to interact with gB and facilitate HSV-1 entry although it has remained unclear whether specific gB-receptor interactions are critical for entry.

Crystal structures of HSV-1 [13] and Epstein–Barr virus (EBV) [14] demonstrated that gB has structural similarity with other viral fusion proteins, such as the G protein of vesicular stomatitis virus (VSV) [15] and gp64 of baculovirus [16], and likely acts as the key herpesviral fusion protein that requires gH/gL as a fusion regulator [17,18,19,20]. However, it is not fully understood how gB and gH/gL interact during viral fusion.

EHV-1 gB was first described in 1985 as antigenically and structurally similar to HSV-1 gB [21]. Later, it was shown that, like gB of other alphaherpesviruses, EHV-1 gB is essential for viral growth and direct cell-to-cell spread. This was deduced from the fact that only single infected cells, but no viral plaques, were observed when EHV-1∆gB was used to infect non-complementing cell lines. A strong indication for its role as a fusogen was also reported; when viral titers of a gB-deficient EHV-1 virus could partly be restored by adding polyethylene glycol to induce fusion [3]. However, there is no data available on the function of EHV-4 gB, the close relative of EHV-1.

EHV-4 (145 kbp) and EHV-1 (150 kbp) both contain 76 unique and highly similar genes. Nucleic acid identity between EHV-4 and EHV-1 genes in general is above 80%, indicating that they are functionally closely related [22,23,24]. However, despite this high genetic similarity, the disease outcomes after infection differ substantially. EHV-4 only induces mild symptoms that are usually limited to upper respiratory tract infections associated with fever and general malaise [25,26]. While also causing respiratory disease, EHV-1 induces more severe clinical disease that includes abortion in pregnant mares [27,28,29] and the so-called EHV-1 myeloencephalopathy (EHM) [30,31]. On the cellular level, numerous differences in viral-cell interaction have been described. For instance, both viruses enter cells trough different pathways. More specifically, EHV-1 enters equine epithelial cells via direct fusion at the plasma membrane, while EHV-4 does so via an endocytic pathway [32].

**Figure 1:**
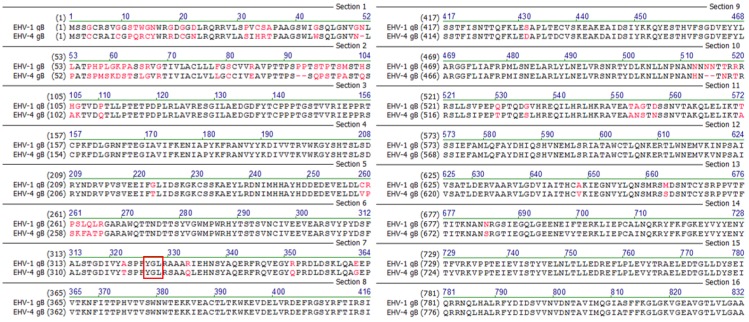
Amino acid sequence alignment of Equine Herpesvirus Type 1 and Type 4 (EHV-1 and EHV-4) glycoprotein B (gB). The putative integrin-binding motif tyrosine-glycine-leucine (YGL) present in the extracellular domains of both gB1 and gB4 (red frame). gB1 and gB4 exhibit 81.1% sequence identity. Sequences were aligned using Vector NTI software (version 9, Invitrogen, Carlsbad, CA, USA, 2004).

The close genetic relatedness allowed us to exchange essential genes between EHV-1 and EHV-4 and to evaluate the effects both* in vitro* and* in vivo* in a way not possible for other members of the subfamily since EHV-1 and EHV-4 naturally infect the same host. We have been interested in exchanging glycoproteins that are part of the cell entry complex between EHV-1 and EHV-4 to further elucidate the process of virus entry [32,33,34,35]. So far, gD was found to play an essential role in determining the cellular tropism of EHV-1 and EHV-4 in culture [33]. gH on the other hand was shown to be responsible for differences in the entry route taken by EHV-1 and EHV-4 [32]. We were interested in exchanging gB to uncover possible functional differences between the two viruses, thereby further elucidating the role of gB in tropism and pathogenicity. gB is highly similar between EHV-1 and EHV-4 and the proteins share an amino acid identity of 81.1% (Figure 1).

gB also contains a putative integrin-binding motif, tyrosine-glycine-leucine (YGL), which is conserved in both EHV-1 and EHV-4, and can potentially interact with α4β7, α4β1, and α9β1 integrins [36]. YGL is also present in the VP4 spike protein of rotaviruses where it mediates cell entry [36]. In a recent study, a similar integrin binding motif, leucine-aspartic acid-isoleucine (LDI), present in EHV-1 gH and interacting with cellular α4β1 integrins, has been implicated in determining the entry pathway taken by EHV-1 in equine cells [32]. Since integrin-binding motifs were shown to have significant roles during viral infection, we addressed the role of YGL-motif during EHV-1 and EHV-4 entry.

Here we show that exchanging gB between EHV-1 and EHV-4 resulted in the generation of stable recombinant viruses; however, a significant attenuation in the case of EHV-4_gB1 was evident.

## 2. Materials and Methods

### 2.1. Viruses

EHV-1 strain Ab4 [isolated from a quadriplegic mare [37] was cloned as a bacterial artificial chromosome (BAC) by replacing the nonessential *gp2* gene with a mini-F plasmid, containing a *chloramphenicol resistance gene* and the enhanced green fluorescence protein (*eGFP*) gene, (Ab4∆gp2) [38,39,40]. The EHV-4 infectious BAC clone was generated by the insertion of a *lox*P-flanked BAC vector into the intergenic region between genes *58* and *59* [41]. Viruses were reconstituted after transfecting BAC DNA into human embryonic kidney (293T) cells, as described earlier [41,42,43]. Supernatant and cells were collected 48 h post-transfection, and high titer stocks of each virus were produced by passaging the transfection product on equine dermal (ED) cells.

### 2.2. Plasmids

Transfer plasmids encoding either EHV-1 or EHV-4 *gB* with a kanamycin resistance (*KanR*) gene were constructed. EHV-1 and EHV-4 *gB* genes were amplified by PCR using primers P1 and P2 or P3 and P4 (Table 1). The PCR products were digested with the restriction enzymes XhoI and XbaI (New England Biolabs, NEB, Schwalbach, Germany) and inserted into the vector pBluescript II KS+ (pKS), resulting in recombinant plasmids pKSgB1 and pKSgB4. To construct pKSgB1-KanR and pKSgB4-KanR, the *KanR gene* was amplified by PCR from plasmid pEPkan-S using primers P5, P6, P7, and P8 (Table 1), digested with the appropriate restriction enzymes, and inserted into pKSgB1 and pKSgB4. Correct amplification and insertion were confirmed by Sanger sequencing (LGC Genomics, Berlin, Germany).

**Table 1:** Oligonucleotide primers used in this study.

Primer	Product	Sequence
P1	gB1	*aat***ctcgag**atgtcctctggttgccgttc
P2	gB1	*aac***tctaga**ttaaaccattttttcatttt
P3	gB4	*aat***ctcgag**atgtccacttgttgccgtgc
P4	gB4	*aca***tctaga**ttaaaccattttttcgcttt
P5	KanR 1	*acc***ggatcc**accgtcgtacgcatcgaaccaggatgacgacgataagtaggg
P6	KanR 1	*ggt***ggatcc**ggtaggcggtgggcaggtgtcaaccaattaaccaattctgattag
P7	KanR 4	*act***ggatcc**acagttgtacgcattgaaccaggatgacgacgataagtaggg
P8	KanR 4	*tgt***ggatcc**agtaggcggcgggcaggtgtcaaccaattaaccaattctgattag
P9	gB1 deletion	agcgctgcgtgagcggcatttacataacctacgaggcgtcacatgtttaataaatattat aggatgacgacgataagtaggg
P10	gB1 deletion	tcacactttgagtacgtgtcataatatttattaaacatgtgacgcctcgtaggttatgta caaccaattaaccaattctgattag
P11	gB4 deletion	agcgctgcgctagcggcatttacataacatacgagacgtcaaatgttaaataaatatttt aggatgacgacgataagtaggg
P12	gB4 deletion	tcaacccacaagtacgtgtcaaaatatttatttaacatttgacgtctcgtatgttatgta caaccaattaaccaattctgattag
P13	gB4 KanR	agcggcgcacagcgctgcgtgagcggcatttacataacctacgaggcgtcatgtccacttgttgccgtgc
P14	gB4 KanR	aaatatgaggtcacactttgagtacgtgtcataatatttattaaacatgtttaaaccattttttcgcttt
P15	gB1 KanR	aacggcgcacagcgctgcgctagcggcatttacataacatacgagacgtcatgtcctctggttgccgttc
P16	gB1 KanR	caaatatgagtcaacccacaagtacgtgtcaaaatatttatttaacatttttaaaccattttttcatttt
P17	gB^Y336A^	ctgtccaccggtgatattgtgtacgcgtctccgttt**GC**cggcctgagggctgccgctcgc aggatgacgacgataagtaggg
P18	gB^Y336A^	gtagctattgtgctctatgcgagcggcagccctcaggccg**GC**aaacggagacgcgtacac caaccaattaaccaattctgattag
P19	Sequencing	ctcggttttccactgtggag
P20	Sequencing	ggtgaatgaggatgaaacct
P21	Sequencing	cgaccacgccaagccccccaac
P22	Sequencing	cggcctcccccactttacccag
P23	Sequencing	atcgaaccacctagaacttg
P24	Sequencing	gtcagctggaactggac
P25	Sequencing	gggcgggagtagcacgtgtt
P26	Sequencing	agccccccaaatgggttgt
P27	Sequencing	ccacggtcatgtcccaagtt
P28	Sequencing	ttctcttcggttttccactg
P29	Sequencing	ttggcaaaaatactaggctt

Restriction enzyme sites are given in lower case bold letters; sequences in italics indicate additional bases which are not present in the EHV-1 or -4 sequence; Underlined sequences indicate the template binding region of the primers for PCR amplification with pEPkan-S; Upper case bold letters indicate the nucleotides that were mutated.

### 2.3. Cells

293T, Rabbit kidney (RK13), Henrietta Lacks (HeLa), African green monkey kidney (Vero), Crandell feline kidney (CrFK) and Madin-Darby canine kidney (MDCK) cells were propagated in Dulbecco’s modified Eagle’s medium (DMEM; Biochrom AG, Berlin, Germany) supplemented with 10% fetal bovine serum (FBS; Biochrom AG), and 1% penicillin-streptomycin. ED, Chinese hamster ovary (CHO)-K1, and CHO cells expressing HevA, HevB, and HevC (CHO-A, CHO-B and CHO-C cells, respectively; a kind gift from Dr. Patricia Spear, Northwestern University, Chicago, IL, USA) were grown in Iscove’s modified Dulbecco’s medium (IMDM; Invitrogen, Paisley, UK) supplemented with 20% FBS, 1% nonessential amino acids (Biochrom AG) and 1% 100 mM sodium pyruvate (Biochrom AG).

For generation of Vero cells, which transiently express EHV-4 *gB* (Vero/gB4), cells were transfected with the vector pcDNA3 (Invitrogen, Paisley, UK) containing *gB4* using Lipofectamine 2000 (Invitrogen, Paisley, UK).

Peripheral blood mononuclear cells (PBMC) were isolated from heparinized blood collected by density gradient centrifugation over Histopaque 1077 (Sigma, St. Louis, MO, USA), following the manufacturer’s instructions. After two washing steps, cells were suspended in RPMI 1640 supplemented with 10% FBS, 0.3 mg/mL glutamine, non-essential amino acids, and 1% penicillin-streptomycin.

### 2.4. BAC Mutagenesis

**Figure 2:**
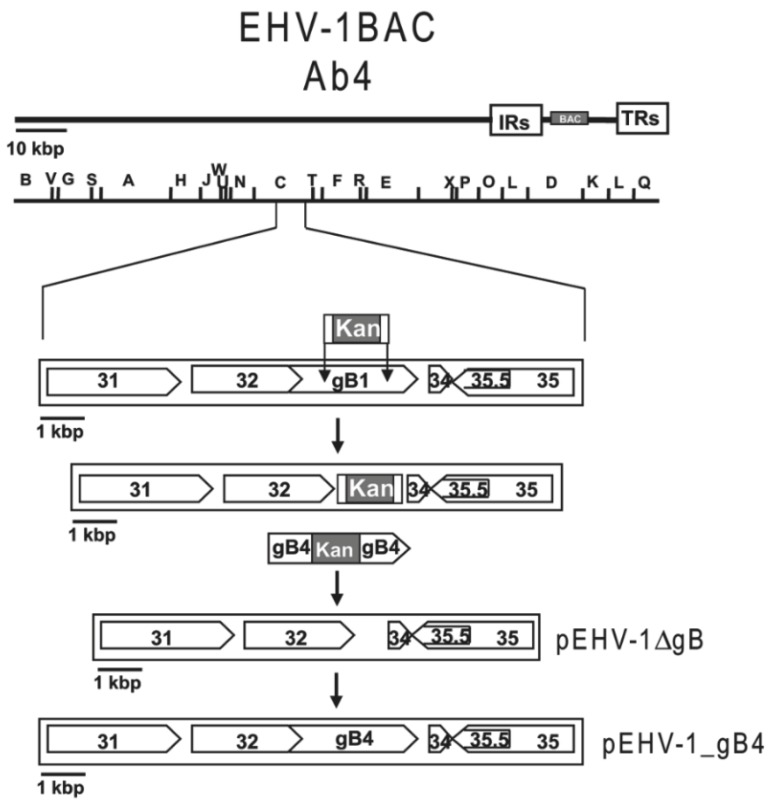
Schematic diagram of the procedures used to construct mutant genomes (EHV-1_gB4 as model). The two unique regions (UL and US) of the EHV-1 genome as well as the terminal and internal repeat sequences (TRS and IRS) are shown, as is the inserted mini-F cassette. PCR products of the kanamycin resistance gene (*KanR*) were transformed into GS1783 containing the EHV-1 bacterial artificial chromosomes (BACs) by electroporation and the first step of the Red recombination method ensued. In the second step of Red recombination, the *KanR gene* was excised, thereby obtaining the pEHV-1∆gB deletion construct. The PCR-amplified gB4Kan was then electroporated into GS1783 harboring pEHV-1∆gB, and, again, two-step Red recombination was performed to obtain the final construct pEHV-1_gB4.

The generated BACs were maintained in *Escherichia coli* (*E. coli*) GS1783 (a kind gift from Greg Smith, Northwestern University, Chicago, IL, USA), which harbor the recombination system of phage λ under the control of a temperature-sensitive repressor [44]. Deletion of the *gB* gene (*UL27*) in EHV-1 and EHV-4 was done by a two-step recombination method (Figure 2) as described before [45]. Briefly, primers P9, P10, P11, and P12 (Table 1) were used to generate homology arms (50 nucleotides) by PCR enabling the substitution of *gB* by *KanR*. PCR products were digested with DpnI in order to remove residual template DNA. The fragments were then transformed into GS1783 cells containing the BACs by electroporation. Kanamycin-resistant colonies were purified and screened by PCR and restriction fragment length polymorphism (RFLP) to detect correctly recombinant clones. Positive clones were subjected to a second round of Red recombination, excising the *KanR* gene, to obtain the intermediate constructs pEHV-1∆gB and pEHV-4∆gB.

The transfer gB1Kan and gB4Kan sequences were amplified by PCR using pKSgB1-KanR and pKSgB4-KanR as templates and primers P13, P14, P15, and P16 (Table 1). PCR products were then electroporated into GS1783 harboring pEHV-1∆gB or pEHV-4∆gB. After selection on LB agar plates containing 34 μg/mL chloramphenicol and 50 μg/mL kanamycin, resistant colonies were purified and screened by PCR and RFLP to detect *E. coli* harboring recombinant pEHV-1_gB4Kan and pEHV-4_gB1Kan. Positive clones were subjected to a second round of Red recombination to obtain the final constructs pEHV-1_gB4 and pEHV-4_gB1.

A point mutation targeting the YGL motif present in EHV-1 gB was generated by introducing alanine into the tyrosine position (EHV-1_gB^Y336A^). Mutants were generated through two-step red recombination using primers P17 and P18 (Table 1) as described above.

The respective genotypes of all the mutants and revertants were confirmed by PCR, RFLP, and Sanger sequencing (primers P19-P29; Table 1; data not shown).

### 2.5. Western Blot Analysis

For Western blot analyses, pellets of infected ED cells were suspended in radioimmunoprecipitation assay (RIPA) buffer (50 mM Tris (pH 7.4), 1% Triton X-100, 0.25% Na-deoxycholate, 150 mM sodium chloride, 1 mM EDTA) with the complete EDTA-free protease inhibitor cocktail (Roche, Basel, Switzerland). Sample buffer (1 M Tris-HCl (pH 6.8), 0.8% sodium dodecyl sulfate (SDS), 0.4% glycerol, 0.15% β-mercaptoethanol, 0.004% bromophenol blue) was added to lysates, the mixture was heated at 95 °C for 5 min, and proteins were separated by 10% SDS-polyacrylamide gel electrophoresis (PAGE) as described before [46]. Expression of gB was detected with anti-EHV-1 gB MAb 3F6, which is also cross-reactive with EHV-4 gB (1:1000) [47]. Rabbit anti-β-actin antibody (1:2000) (Cell signaling Technologies, Danvers, MA, USA) was included as a loading control. Goat anti-mouse or goat anti-rabbit (1:10,000) IgG peroxidase conjugates (Southern Biotech, Birmingham, AL, USA) were used as secondary antibodies. Reactive bands were visualized by enhanced chemiluminescence (ECL Plus; Amersham, GE Healthcare, Piscataway, NJ).

### 2.6. Virus Growth Assays

To determine differences in viral replication in culture, single step growth kinetics for EHV-1 and multi-step growth kinetics for EHV-4 were conducted as described before [34]. Briefly, confluent ED cells were infected with a multiplicity of infection (MOI) of 1 in case of EHV-1 parental virus, EHV-1_gB4 and EHV-1 revertant. An MOI of 0.1 was used for EHV-1_ gB^Y336A^ in comparisons with parental EHV-1. In case of parental EHV-4, EHV-4_gB1, and revertant EHV-4, an MOI of 0.01 was used. After 1 h, cells were washed and treated with citrate buffer (pH = 3). Infected cells and supernatant were collected separately for EHV-1 and combined for EHV-4 at the indicated times post infection (p.i.), and stored at −80 °C. Viral titers were determined on ED cells.

Plaque size measurements were conducted by infecting ED cells for 1 h at 37 °C with an MOI of 0.01, followed by removal of the virus suspension and overlay with DMEM containing 0.5% methylcellulose (Sigma). After 72 h.p.i, the diameters of 100 fluorescent plaques for each virus were measured using ImageJ software vl.32j (National Institutes of Health, Bethesda, MD, USA, 2004) (http://rsb.info.nih.gov/ij/). The obtained values were normalized and compared to the values of parental viruses, which were set to 100%. Three independent experiments were used to calculate average plaque sizes and standard deviations.

### 2.7. Virus Infection Assay

For evaluating efficiency of replication and cell tropism, confluent monolayers of different cell types were infected with an MOI of 0.1 of the respective viruses. Cells were then washed and overlaid with the appropriate medium. After 48 h p.i, cells were inspected with immunofluorescence microscope (Zeiss Axiovert, Jena, Germany) and pictures were taken with an Axiocam CCD camera (Zeiss, Jena, Germany).

### 2.8. Flow Cytometry

One million PBMC were incubated with EHV-1 and the engineered recombinant viruses at an MOI of 1 for 48 h at 37 °C. After incubation, the percentage of infected cells was determined by measuring eGFP expression with a FACSCalibur flow cytometer (BD Biosciences, San Jose, CA, USA).

### 2.9. Pharmacological Inhibitors

Cells were pretreated with different drugs for 60 min as described before [32], and infected with parental and recombinant viruses using an MOI of 0.05 for 24 h in the presence of the drugs. Cells were then trypsinized and washed twice with PBS. After centrifugation, cells were resuspended in PBS, and 10,000 cells were analyzed for eGFP expression with a FACSCalibur flow cytometer. The drug concentrations used were 100 μg/mL genistein (Sigma) dissolved in DMSO, 10 μg/mL chlorpromazine (Sigma) in PBS, and 80 μM dynasore (Sigma) in DMSO.

### 2.10. Statistical Analysis

Statistical analyses (described in context) were performed using GraphPad PRISM (Version 5, GraphPad Software Inc., La Jolla, CA, USA, 2007). Normally distributed datasets, determined with the Shapiro–Wilks test, were analyzed with one-way ANOVA. Datasets that were not normally distributed were analyzed with Kruskal–Wallis one-way analysis of variance for two or more samples that are independent or the Friedman test for repeated measures.

## 3. Results

### 3.1. gB Expression by the Recombinant Viruses

**Figure 3:**
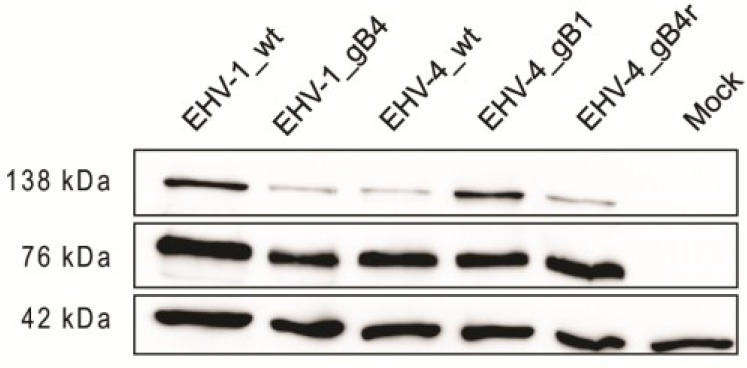
Expression of gB in virus-infected cells. Cell lysates were prepared from infected equine dermal (ED) cells and proteins were separated under reducing conditions by sodium dodecyl sulfate (SDS)-10%-polyacrylamide gel electrophoresis (PAGE). The blots were incubated with anti-gB monoclonal antibody (MAb)-3F6 and bound antibody detected with anti-mouse IgG peroxidase conjugate. EHV-1, EHV-4 and related recombinant virus proteins with an apparent molecular weight of approximately 130 and 76 kD were detected that are not present in mock-infected cells. Rabbit anti-β-actin antibody was used as a loading control (molecular weight of approximately 42 kDa).

To determine whether gB was properly expressed by the recombinant viruses, ED cells were infected with parental, recombinant and revertant viruses. Cell lysates were then collected and subjected to Western blot analysis. For parental EHV-1, EHV-4 and related recombinant viruses, proteins with molecular weights of approximately 138 and 76 kD were detected that were not present in mock-infected cells (Figure 3). This is in accordance with previous reports where a partially glycosylated precursor of approximately 138 kD and a fully glycosylated subunit of gB of 75–77 kD were reported to be specifically recognized by MAb 3F6 [48]. These experiments confirmed that all generated recombinant and revertant viruses expressed gB as expected.

### 3.2. EHV-4 gB Is Essential for Viral Replication

**Figure 4:**
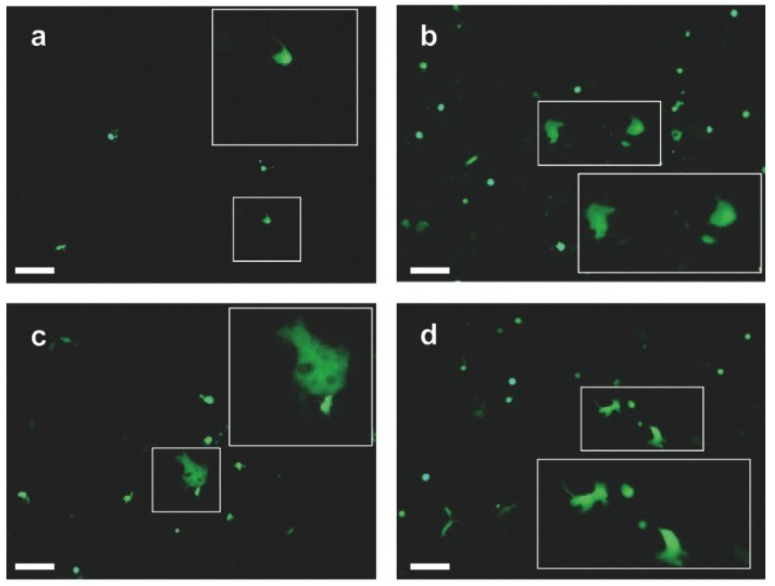
Transfection of African green monkey kidney (Vero) cells with EHV-4ΔgB. EHV-4ΔgB DNA was transfected in Vero cells (**a**) and Vero/gB4 cells (**b**). Also, parental EHV-4 DNA was transfected in both Vero (**c**) and Vero/gB4 cells (**d**). After 48 h of incubation, cells were inspected with an epifluorescent microscope (Zeiss Axiovert, Jena, Germany) and images were taken with a CCD camera (Zeiss Axiocam, Jena, Germany). The bar represents 100 μm and the white frames contain magnified inserts of the selected areas. Plaque formation of the gB-negative EHV-4 mutant was only evident on Vero/gB4 but not parental Vero cells.

pEHV-4∆gB DNA was transfected into Vero cells and the cells were monitored for 48 h. Only single infected cells (eGFP-positive/fluorescent cells) could be detected of which the number did not increase over time (Figure 4a). In contrast, Vero cells transfected with parental EHV-4 DNA showed syncytium formation after 48 h (Figure 4c). When Vero cells, which were transfected with gB4 as described previously for gH [35], were transfected with EHV-4∆gB (Figure 4b), syncytium formation with a morphology similar to that induced by parental EHV-4 (Figure 4d) could be seen.

### 3.3. Virus Growth in Culture

**Figure 5:**
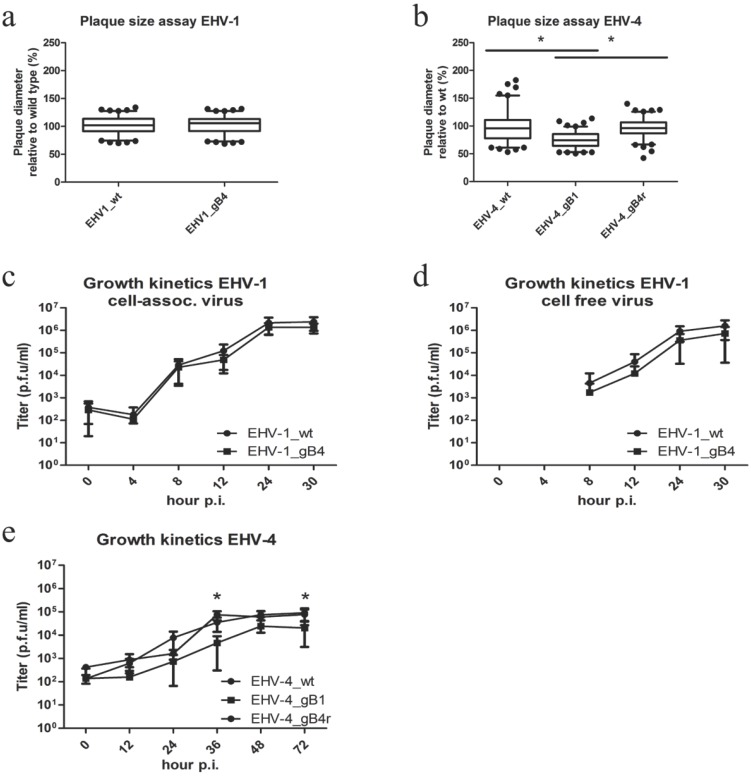
Growth characteristics of parental and recombinant EHV-1 and EHV-4 in cell culture. ED cells were infected with the respective viruses at a multiplicity of infection (MOI) of 0.01. Means ± SD of diameters of 100 plaques measured for each virus are shown. The plaque diameter of parental viruses was set to 100%. No significant differences (one-way ANOVA; *p* > 0.05) between parental EHV-1 and EHV-1_gB4 were obvious (**a**). A significant reduction (one-way ANOVA; *p* < 0.05) of plaque size for EHV-4_gB1 was evident when compared to parental and revertant virus. Means ± SD of diameters of 100 plaques measured for each virus are shown. The plaque diameter of parental viruses was set to 100% (**b**). For single step growth kinetics of EHV-1 recombinant viruses, ED cells were infected at an MOI of 1 (**c**,**d**), followed by citrate treatment (pH = 3) to remove remaining extra-cellular virions. Infected cells (**c**) and supernatants (**d**) were separately collected and virus titers were determined at the indicated times post-infection (p.i). The data presented are means ± SD of three independent measurements. No significant differences were measured for the EHV-1 recombinant viruses when compared to the parental viruses (Friedman test-Dunn’s multiple comparison test; *p* > 0.05). (**e**) For multi-step growth kinetics of EHV-4_gB1, ED cells were infected at MOI of 0.01, followed by washing. Infected cells and supernatants were collected and virus titers were determined at the indicated times p.i. The data presented are means ± SD of three independent measurements. A significant decrease was measured for EHV-4_gB1 at several time points (*) when compared to the parental and revertant viruses (Friedman test–Dunn’s multiple comparison test; *p* < 0.05).

No significant differences were observed for any of the EHV-1 recombinants when compared to the parental virus (Figure 5a,c,d). We therefore concluded that the growth properties of recombinant EHV-1 in culture were not significantly affected by the exchange with EHV-4 gB.

In the case of EHV-4_gB1, however, significantly reduced plaque sizes were seen compared to parental and revertant viruses (Figure 5b). Similarly, EHV-4_gB1 also showed a significantly reduced growth rate at several time points as evidenced by the growth kinetics (Figure 5e).

### 3.4. gB Has No Role in Determining the Host Range of EHV-1 and EHV-4 in Culture

**Figure 6:**
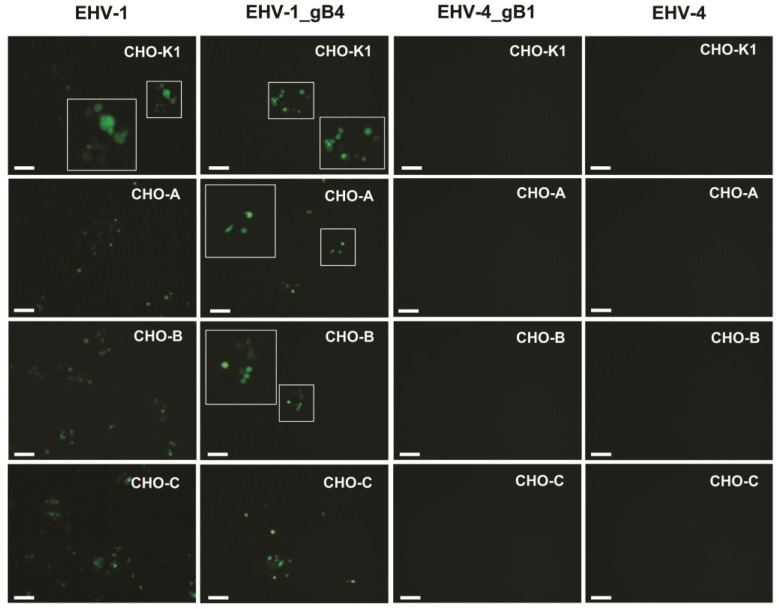

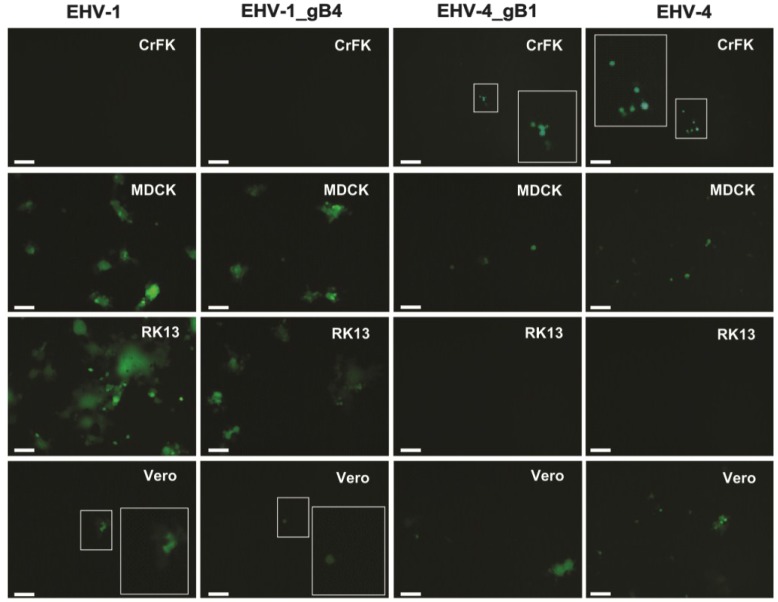
The role of gB in EHV-1 cellular tropism. Chinese hamster ovary (CHO)-K1, CHO-A, CHO-B, CHO-C, Crandell feline kidney (CrFK), Madin-Darby canine kidney (MDCK), Rabbit kidney (RK13) and Vero cells were infected at an MOI of 0.1 with the parental EHV-1 and EHV-1_gB4, all of which express eGFP. At 24 h p.i., cells were inspected with a fluorescent microscope (Zeiss Axiovert, Jena, Germany) and images were taken with a CCD camera (Zeiss Axiocam, Jena, Germany). The bar represents 100 μm and the white frames contain magnified inserts of the selected areas. CHO-K1, CHO-A, CHO-B and CHO-C, and RK13 cells were highly resistant and MDCK virtually resistant to parental and recombinant EHV-4 infection. In addition, CrFK were highly resistant and Vero cells virtually resistant to parental and recombinant EHV-1 infection.

EHV-1 can replicate and spread in many cell lines from equine and other origin [49]. In contrast, EHV-4 appears to be restricted mainly to equine cells and replicates poorly in only few cell lines, such as Vero cells [43]. In order to investigate whether gB plays a role in determining host range, as already shown for gD [33], several cell lines that are only permissible for either EHV-1 or EHV-4 were infected. Contrary to the exchange of gD between the two viruses, no changes in permissiveness were seen after exchanging gB between EHV-1 and EHV-4 (Figure 6; Table 2).

**Table 2:** The role of gB in EHV-1 cellular tropism.

Cell line	EHV-1	EHV-1 gB4	EHV-4	EHV-4 gB1
CHO-K1	+	+	−	−
CHO-A	+	+	−	−
CHO-B	+	+	−	−
CHO-C	+	+	−	−
CrFK	−	−	+	+
MDCK	+	+	+	+
RK13	+	+	−	−
Vero	+	+	+	+

### 3.5. The Integrin-Binding Motif YGL Is Not Involved in EHV-1 Entry

The integrin-binding motif YGL was predicted with the I-TASSER server and is present in the extracellular domain of gB (http://zhang.bioinformatics.ku.edu/I-TASSER/). The motif is conserved in both EHV-1 and EHV-4. The YGL motif was mutated into AGL in EHV-1 (EHV-1_gB^Y336A^) to evaluate its importance for cell entry and determining the cell entry pathway.

**Figure 7:**
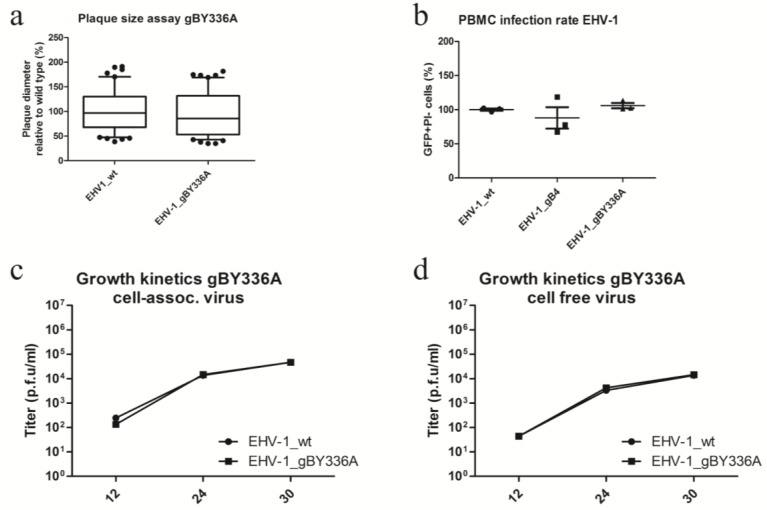
Characterization of EHV-1_gBY336A. (**a**) ED cells were infected with the respective viruses at an MOI of 0.01. Means ± SD of diameters of 100 plaques measured for each virus are shown. The plaque diameter of parental viruses was set to 100%. No significant differences (one-way ANOVA; *p* > 0.05) between parental EHV-1 and EHV-1_gBY336A were evident; (**b**) One million peripheral blood mononuclear cells (PBMC) were incubated with parental and recombinant EHV-1 viruses at an MOI of 1 for 24 h at 37 °C. After incubation, the percentage of infected cells was determined by flow cytometry. The rate of infection of parental virus was set to 100%. All data represent the means ± SD of three independent experiments (Kruskal–Wallis one-way analysis of variance; *p* > 0.05). For single-step growth kinetics of EHV-1 recombinant viruses, ED cells were infected at an MOI of 0.1 (**c,d**), followed by citrate treatment (pH = 3) to remove remaining extracellular virions. Infected cells (**c**) and supernatants (**d**) were separately collected and virus titers were determined at the indicated times p.i. The data presented are means ± SD of three independent measurements. No significant differences were detectable for the EHV-1 recombinant viruses when compared to the parental viruses (Friedman test–Dunn’s multiple comparison test; *p* > 0.05).

In previous studies, function-blocking MAbs α4β1 and α4β7 were used to investigate the role of integrins during entry into PBMC, ED and fetal horse kidney (FHK) cells. It was shown that blocking these potential receptors for EHV-1 and EHV-4 had no effect on their growth in culture or their ability to infect PBMC [35]. However, this does not exclude the possibility that the YGL motif could have an effect on viral growth and infection rates, since other unknown binding partners could be involved. However, no changes could be seen for either viral growth in culture (Figure 7a,c,d) or infection rates in PBMC (Figure 7b) between parental EHV-1 and EHV-1_gB^Y336A^.

### 3.6. gB and YGL Do Not Play a Role in Determining the Cell Entry Pathway

To investigate whether gB or the YGL motif have an effect on the viral entry pathways used by EHV-1, we conducted inhibitor studies using drugs that target various cellular functions associated with viral cell entry as described previously for gH [32].

Caveolae are plasma membrane invaginations that play an important role in cellular uptake of EHV-4 or EHV-1 after replacing authentic gH1 with EHV-4 gH [32]. Caveolae-mediated uptake depends on dynamin II [50], which can be inhibited by dynasore [51], and tyrosine kinases, which can be inhibited by genistein [52]. Chlorpromazine affects Clathrin-mediated endocytosis through the inhibition of Clathrin adaptor protein 2 assembly [53]. Since this drug does not affect the uptake of EHV-1 and EHV-4 [32], it was used as a negative control.

**Figure 8:**
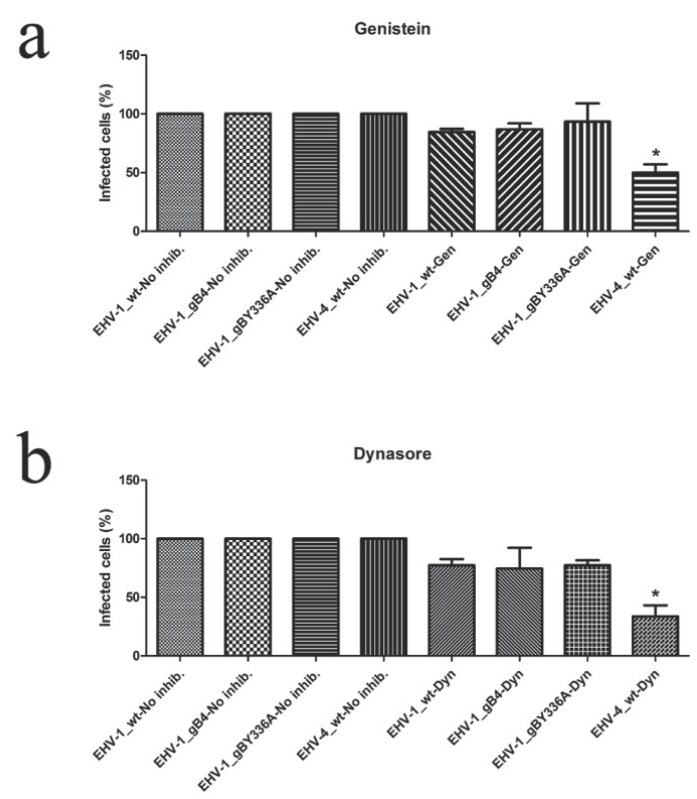

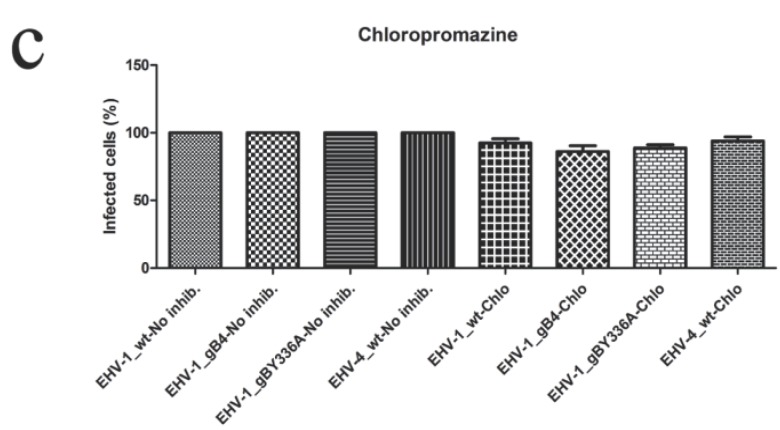
Inhibition of cell entry. Equine dermal (ED) cells were treated with the inhibitors genistein (Gen; **a**), dynasore (Dyn; **b**) and chlorpromazine (Chlo; **c**), as indicated, before infection with parental or recombinant viruses at an MOI of 0.05. At 24 h p.i., cells were washed and the percentage of infected cells was determined by flow cytometry. The percentage of infection of the parental virus was set to 100%. The percentage of infection (eGFP^+^) in the absence of inhibitors was set to 100%. All data represent the means ± SD of three independent experiments. Significant decreases (*) were only seen for EHV-4_wt in the presence of dynasore and genestein (Kruskal–Wallis one-way analysis of variance; *p* < 0.05). However, no significant differences in infection rate were seen between parental and recombinant viruses in the different settings (Kruskal–Wallis one-way analysis of variance; *p* > 0.05).

Contrary to the differences in cell entry pathway seen when exchanging gH, no significant differences could be seen between parental EHV-1 and EHV-1_gB4 or EHV-1_gB^Y336A^ after using different inhibitors. In the case of EHV-4_gB1 as well as parental EHV-4, the number of infected cells was significantly reduced when treated with either genistein or dynasore. These data clearly indicated that gB has no role in routing the entry pathway of EHV-1 (Figure 8), but is rather required for entry steps after receptor binding.

## 4. Discussion

For several alphaherpesviruses, including EHV-1, it has been shown that gB is essential for infection [7,54,55,56,57,58]. More specifically, gB plays an important role in the cell-to-cell spread of alphaherpesviruses [2,3,4,5,6]. In the present study, we have addressed the importance of gB for EHV-4 replication. We have shown that the number of infected cells after transfection with pEHV-4∆gB did not increase over time. This suggests that EHV-4 is not able to either be released from infected cells or enter uninfected cells without the help of gB. The gB-deleted virus was only able to induce syncytium formation when grown in pcDNAgB4-transfected Vero cells, in other words when the glycoprotein was provided *in trans*. It has been shown that gB is involved in virus maturation and egress from the infected cells for different herpesviruses including HSV-1 and Kaposi’s sarcoma-associated herpesvirus [59,60]. EHV-4 gB may also play a role in viral egress, including first or secondary envelopment or virion release into the extracellular space. However, we view an involvement of EHV-4 gB in egress as unlikely as those of other varicelloviruses are not required in the process either. Rather, we presume an essential role in virus entry and cell-to-cell spread but the exact role and the mechanism are not known at present and need to be further investigated. Taken together, the essentiality of gB functions during different virus replication steps is not unique to EHV-4 as all of the gB homologues across all subfamilies of the *Herpesviridae* studied until now have been shown to play similar roles.

The replacement of gB1 by gB4 in EHV-1 did not lead to significant changes in viral growth in culture. However, the EHV-4_gB1 recombinant exhibited a markedly reduced growth defect as evidenced by impaired viral cell-to-cell spread and reduced growth kinetics. This was particularly surprising since no apparent growth defect was seen in culture during previously conducted experiments where gB were exchanged between alphaherpesviruses of different natural hosts [61]. The growth defect of EHV-4_gB1 could be caused by a structural incompatibility of gB1 that EHV-4 was unable to compensate for. Alternatively, gB4 might have an additional role required for EHV-4 replication but not fulfilled by gB1. In a previous study, where gB of HSV-1 was replaced with gB of Saimiriine Herpesvirus 1 (SaHV-1), it was shown that HSV-1 gB possessed and additional function lacking for SaHV-1 gB. Loss of functional interaction with PIRLα was reported for SaHV-1 despite sequence alignment suggesting that the interaction site is conserved [62].

Therefore, it would be interesting to further elucidate the structural differences between gB1 and gB4 and identify putative viral interaction partners of gB. After binding of gD to cellular receptors it activates gH-gL complex, which in turn primes gB for fusion. Perhaps, the authentic EHV-4 gH-gL complex could not prime gB1 as efficiently as the native gB4 for fusion. It will, therefore, be interesting to examine whether a triple mutant,* i.e*., EHV-4 harboring gH1-gL1-gB1 and *vice versa*, would lead to any changes of the virus’ ability to effectively replicate.

Recently it was shown that EHV-1 gD, besides its primary interaction partner MHC class I, can bind to a wide array of receptors and that it is responsible for determining the host range of the virus. The case is less clear for EHV-4 gD, which also uses MHC class I as an entry receptor and likely another molecule to enter, e.g. Vero cells [33]. In the current study, we showed that exchanging gB has no effect on the entry of the recombinant viruses into the selected cell lines, suggesting that gB is not important in determining cellular host range. It seems likely that, in contrast to HSV-1 [10,11,12], gB of EHV-1 or EHV-4 does not need to bind to different cellular receptors to facilitate fusion, that gB binding is not important for cellular tropism, or that gB and receptor interaction are not absolutely required for entry. However, this conclusion does not exclude the role of gB, together with gC, to bind cell surface heparan sulfate and help the attachment of virions to cells during the initial events of infection [34].

Disrupting the integrin-binding motif YGL in gB1 did not have any effect on virus growth in culture. Furthermore, YGL apparently does not play a decisive role in determining the cell entry pathway of EHV-1 or EHV-4. These results are in accordance with previous work where binding between the integrin-binding motif YGL and selected known ligands α4β1 or α4β7 was mitigated using blocking antibodies. These experiments revealed that YGL does not need to interact with its known binding partners for infection [35]. From these results, we concluded that gB-integrin interaction does not play an important role in cell entry or determining the cell entry pathway; however, it may have a role in signaling transduction that might be needed during other steps of virus replication. Furthermore, these data do not necessarily mean that no interaction occurs between EHV-1 and the respective integrins. The integrins may serve as a receptor and/or co-receptor for viral entry and their blockade may not have a measurable effect on virus infection, especially, if alternative receptors exist.

Due to its importance in cell-to-cell spread [6], gB would be an interesting target for future research on the spread of EHV-1 between infected PBMC and endothelial cells (EC). This process enables infection of EC even in the presences of neutralizing antibodies, causing vascular lesions and secondary hypoxic degeneration of affected tissues [24,63].

In summary, the replacement of gB1 by gB4 in EHV-1 did not lead to any significant changes in viral growth in culture compared to EHV-1. However, EHV-4 seems to be unable to fully compensate the structural changes introduced by the replacement of gB4 with gB1 with respect to replication in culture. Nonetheless, the generation of a stable EHV-1_gB4 recombinant virus gives us the tools to address viral entry and spread of EHV-1 and EHV-4 in other cell types in the future.
